# Engineering *Escherichia coli* to increase plasmid DNA production in high cell-density cultivations in batch mode

**DOI:** 10.1186/1475-2859-11-132

**Published:** 2012-09-19

**Authors:** Gheorghe M Borja, Eugenio Meza Mora, Blanca Barrón, Guillermo Gosset, Octavio T Ramírez, Alvaro R Lara

**Affiliations:** 1 Departamento de Medicina Molecular y Bioprocesos, Col. Chamilpa, CP 62210, Cuernavaca, Morelos, Mexico; 2 Departamento de Ingeniería Celular y Biocatálisis, Instituto de Biotecnología, Universidad Nacional Autónoma de México, Avenida Universidad 2001, Col. Chamilpa, CP 62210, Cuernavaca, Morelos, Mexico; 3 Departamento de Microbiología, Escuela Nacional de Ciencias Biológicas, Instituto Politécnico Nacional, Prol. de Carpio y Plan de Ayala s/n, Col. Santo Tomás, CP 11340, Del. Miguel Hidalgo México, DF, Mexico; 4 Departamento de Procesos y Tecnología, Universidad Autónoma Metropolitana-Cuajimalpa, Artificios No. 40, Col. Miguel Hidalgo, Del. Álvaro Obregón, México, DF, CP 01120, México

**Keywords:** Plasmid DNA, DNA vaccines, Overflow metabolism, *E. coli*, Batch cultivation, Acetate

## Abstract

**Background:**

Plasmid DNA (pDNA) is a promising molecule for therapeutic applications. pDNA is produced by *Escherichia coli* in high cell-density cultivations (HCDC) using fed-batch mode. The typical limitations of such cultivations, including metabolic deviations like aerobic acetate production due to the existence of substrate gradients in large-scale bioreactors, remain as serious challenges for fast and effective pDNA production. We have previously demonstrated that the substitution of the phosphotransferase system by the over-expressed galactose permease for glucose uptake in *E. coli* (strain VH33) allows efficient growth, while strongly decreases acetate production. In the present work, additional genetic modifications were made to VH33 to further improve pDNA production. Several genes were deleted from strain VH33: the *recA*, *deoR*, *nupG* and *endA* genes were inactivated independently and in combination. The performance of the mutant strains was evaluated in shake flasks for the production of a 6.1 kb plasmid bearing an antigen gene against mumps. The best producer strain was cultivated in lab-scale bioreactors using 100 g/L of glucose to achieve HCDC in batch mode. For comparison, the widely used commercial strain DH5α, carrying the same plasmid, was also cultivated under the same conditions.

**Results:**

The various mutations tested had different effects on the specific growth rate, glucose uptake rate, and pDNA yields (Y_P/X_). The triple mutant VH33 Δ (*recA deoR nupG*) accumulated low amounts of acetate and resulted in the best Y_P/X_ (4.22 mg/g), whereas Y_P/X_ of strain VH33 only reached 1.16 mg/g. When cultivated at high glucose concentrations, the triple mutant strain produced 186 mg/L of pDNA, 40 g/L of biomass and only 2.2 g/L of acetate. In contrast, DH5α produced only 70 mg/L of pDNA and accumulated 9.5 g/L of acetate. Furthermore, the supercoiled fraction of the pDNA produced by the triple mutant was nearly constant throughout the cultivation.

**Conclusion:**

The pDNA concentration obtained with the engineered strain VH33 Δ (*recA deoR nupG)* is, to the best of our knowledge, the highest reported for a batch cultivation, and its supercoiled fraction remained close to 80%. Strain VH33 Δ (*recA deoR nupG*) and its cultivation using elevated glucose concentrations represent an attractive technology for fast and efficient pDNA production and a valuable alternative to fed-batch cultivations of commercial strains.

## Background

Plasmid DNA (pDNA) is an attractive alternative for immunization and gene therapy against many infectious, genetic and acquired diseases [1]. The common host for pDNA production is the bacterium *Escherichia coli*. Several *E. coli* strains have been reported for pDNA production, such as DH5α [2-4], DH5 [5], DH1 [6,7], JM108 [8]; SCS1-L [9] and DH10B [10]. Most of the strains used for pDNA production are selected by its previous use in laboratory-scale protocols [11,12] and may be not suitable for process-like conditions. For example, the typical challenges for high cell-density cultivations (HCDC) of *E. coli* remain as obstacles for the fast and efficient production of pDNA. Among them, aerobic acetate production is an important drawback, since it causes a loss of productivity and waste of carbon source [13]. Aerobic acetate production -known as overflow metabolism- results from an imbalance between glycolysis and tricarboxylic acids cycle [13,14]. Some of the strains commonly used for pDNA production present elevated overflow metabolism, including *E. coli* DH5α and DH1 [9]. While the conventional way of avoiding overflow metabolism is reducing the glucose uptake in the so called fed-batch mode, the constant supply of glucose to the bioreactor requires additional equipment, results in a decrease of growth rate and frequently causes substrate gradients at the feeding zone in production bioreactors that trigger undesirable physiological effects [15-17]. We have previously demonstrated that the substitution of the natural glucose transport system (PTS) by a constitutively expressed galactose permease under the strong *trc* promoter in *E. coli* allows efficient growth by reducing the glucose uptake rate and consequently decreasing acetate production [18,19]. The modified strain, named VH33, has been tested for pDNA production using high initial glucose concentrations in order to reach high cell-densities in batch mode, yielding the double of pDNA per gram of cell (Y_p/x_) than the parental strain, W3110 [20]. In order to increase carbon availability for nucleotide synthesis, the *pykA* gene (codifying for pyruvate kinase A) was inactivated in VH33, which resulted in a further increase of 70% of Y_p/x_[21]. The possibility of cultivating VH33 strains and derivatives at high cell-density in batch mode is a simple and valuable alternative to fed-batch mode for the fast and efficient production of pDNA both, at early-stages of product development and at technical scale.

Notwithstanding the higher production of VH33 and VH33 Δ*pykA*, compared to W3110, its production levels remain low if compared to commercial strains like DH5α. In the present work, the genes *endA*, *recA*, *deoR* and *nupG* were inactivated in strain VH33 independently and in combination, in order to increase pDNA yields. The best engineered strain was cultivated in batch mode using 100 g/L of initial glucose to attain high cell-densities.

## Results and discussion

### Evaluation of the engineered strains in shake flasks

**Figure 1:**
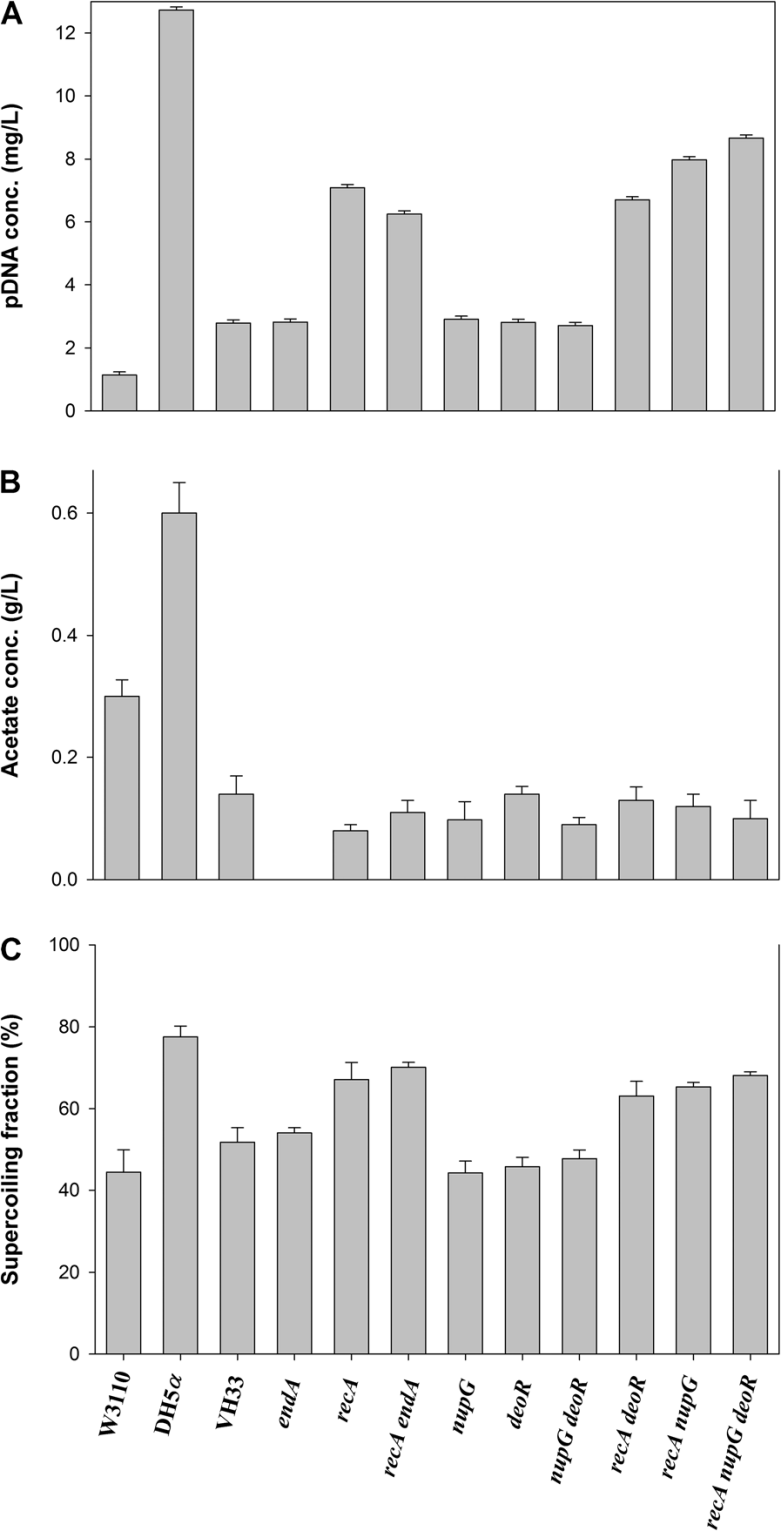
**pDNA concentration (A), Acetate concentration (B) and pDNA supercoiled fraction (C) in shake flask cultivation of the different strains evaluated.** Error bars show the standards deviation between triplicates.

Initially, the growth profiles and pDNA yields of strains W3110 and VH33 were evaluated in shake flasks as described in the Materials and Methods section. Due to the good productivity and wide use of strain DH5α, it was also evaluated and used for comparison. When grown in shake flask, W3110 reached 1.14 ± 0.10 mg/L of pDNA (Figure 1A) and produced 0.32 ± 0.03 g/L of acetate (Figure 1B). Strain VH33 produced 2.78 ± 0.10 mg/L of pDNA and only 0.14 ± 0.01 g/L of acetate (Figure 1A and 1B). The commercial strain DH5α produced 12.73 ± 0.10 mg/L of pDNA, and accumulated a remarkably high amount of acetate, reaching up to 0.62 ± 0.04 g/L (Figure 1B). Regarding the supercoiled fraction, under shake flask conditions, 50% of the pDNA produced by strains W3110 and VH33 was supercoiled, whereas for DH5α cultivations, the supercoiled fraction was around 80% (Figure 1C).

A series of VH33-based mutants were obtained and tested. First, mutations aimed at increasing the plasmid stability were performed. The gene *end A*, coding for a type I endonuclease was deleted, since it has been proposed that such a mutation increases the stability of pDNA [22,23]. As shown in Figure 1, neither the amount of pDNA nor the pDNA supercoiled fraction produced by VH33 Δ *endA* changed with respect to VH33, and no acetate was detected. These results imply that no relevant pDNA degradation occurs intracellularly in *E. coli*, and that the positive effect of *endA* deletion could be seen particularly during downstream operations, although this was not tested.

A second target gene was the *recA*, that codes for recombinase A. An important cause of plasmid instability is the formation of plasmid oligomers, which originates cells with low copy number. Oligomers can be formed by homologous recombination. In *E. coli*, the RecBCD and a variation of RecF pathways are responsible for recombination [24]. The latter requires the products of *recArecFrecJ, recO* genes [25,26]. Recombination via RecBCD and RecF pathways is inactivated by mutations in *recArecBrecC* and *recD*[27]. The formation and breakdown of oligomers is blocked by mutations in *recA* or *recF*[28]. RecA has also a proteolytic activity that activates the Cop protein, a repressor of plasmid replication [27]. In general, it has been reported that *recA* mutants display higher stability [29] and often show a higher pDNA production than parental strains [11,22,30].

As shown in Figure 1A, VH33 Δ *recA* produced 7.09 ± 0.10 mg/L of pDNA, which is 6.2 times more pDNA than W3110 and 2.5 times more than VH33. Moreover, VH33 Δ *recA* retained a low overflow metabolism, since the acetate accumulated was only 0.08 ± 0.01 g/L (Figure 1B). Furthermore, the *recA* mutation resulted in a pDNA supercoiled fraction of 68 ± 8% (Figure 1C). It has been shown that RecA protein participates in the regulation on toposiomerase A gene (*topA*) [31]. Therefore, it is possible that *recA* mutants display a higher topoisomerase activity, which helps to explain the effect observed in VH33 Δ *recA*. Additionally, a double mutant VH33 Δ (*recA endA*) was obtained in order to evaluate a possible synergistic effect of both mutations. A shown in Figure 1, the double mutant produced slightly less pDNA (6.25 ± 0.10 mg/L) than the single mutants, whereas the pDNA supercoiled fraction and acetate accumulation was similar to VH33 Δ *recA*.

A second group of mutations were performed to increase the synthesis of nucleotides, since the availability of such building blocks could be a limiting factor for pDNA synthesis in *E. coli*. The target genes were *deoR* and *nupG*. The *deoR* gene codes for a protein that represses the expression of several genes of the *deo* operon that code for enzymes needed for deoxynucleotide synthesis. Strains lacking *deoR* display a higher level of thymine phosphorylase, phosphopentamutase and deoxyaldolase [32]. Therefore, it was expected that *deoR* mutants could produced more pDNA. Yet, no change with respect to VH33 was observed in pDNA production or acetate accumulation; whereas pDNA supercoiled fraction was lower when VH33 Δ *deoR* was evaluated (Figure 1C). The *nupG* gene codes for a protein involved in nucleotide transport and catabolism, participating in the regulation of genes involved in nucleotide synthesis. It has been shown that *nupG* mutants can produce significantly more purine nucleosides than parental strains [33]. Nevertheless, when *nupG* was deleted in VH33, no change in pDNA production was observed (Figure 1A). Moreover, when both *deoR* and *nupG* mutations were combined in VH33, no positive effect was seen (Figure 1A). A double mutant VH33 Δ (*recA deoR*) was obtained. This strain produced 12% more pDNA (7.98 ± 0.10 mg/L) than VH33 Δ *recA*. In contrast, the double mutant VH33 Δ (*recA nupG*) produced the same amount of pDNA than VH33 Δ *recA* (Figure 1A). Finally, all the three mutations were incorporated in VH33. The triple mutant VH33 Δ (*recA deoR nupG*) produced 22% more pDNA (8.67 ± 0.10 mg/L) than VH33 Δ *recA*, 300% more than VH33 and 760% more than W3110 (Figure 1A). This implies that not only the deletion of genes involved in nucleotide catabolism are necessary to increase pDNA production, but also increasing the plasmid stability is needed to see a positive effect. Another important result is that the triple mutant strain maintained a very low overflow metabolism and that the pDNA produced was supercoiled in 70 ± 5% (Figure 1C).

**Figure 2:**
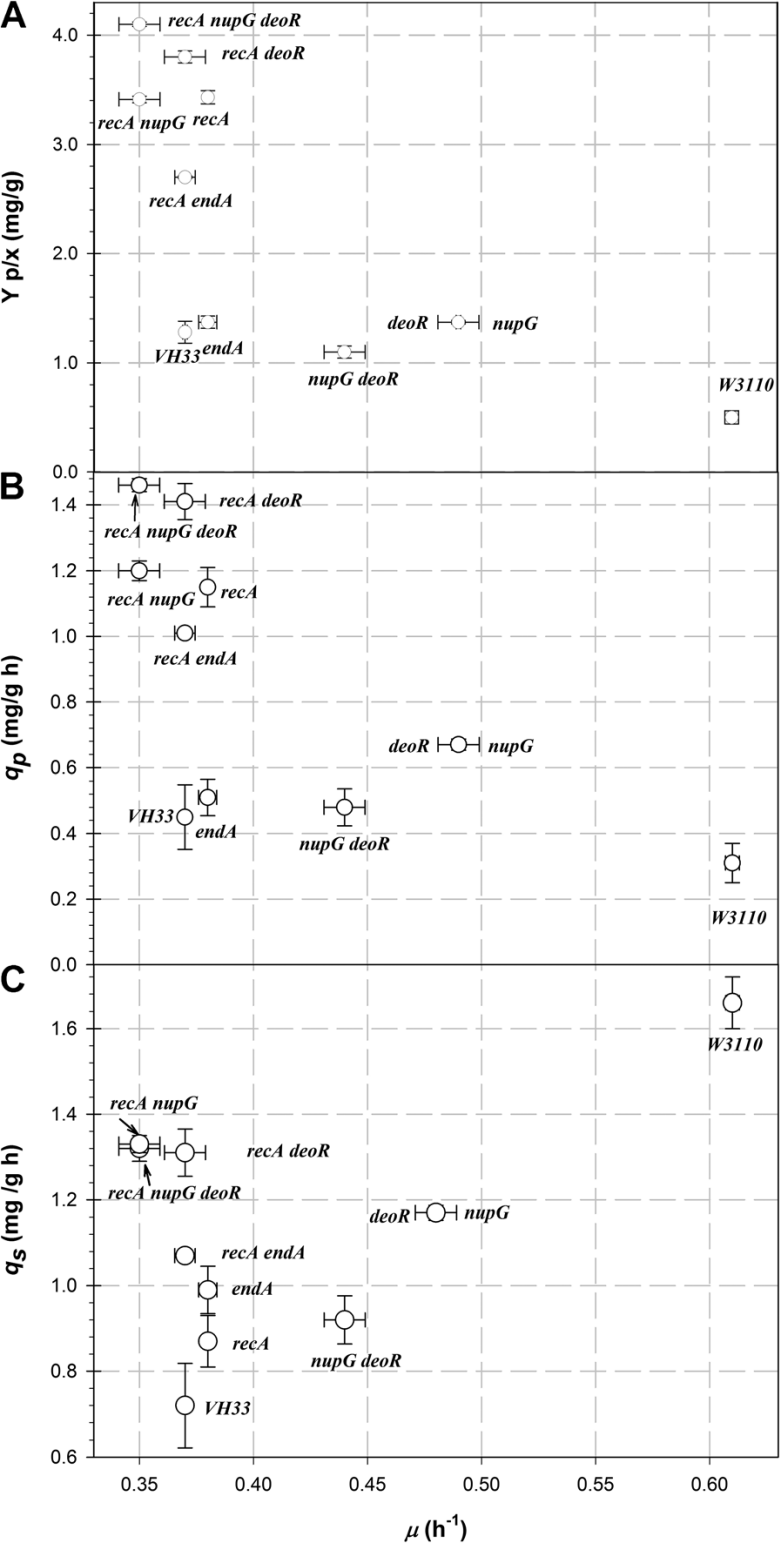
**pDNA yield on biomass (A), specific pDNA production rate (B) and specific glucose uptake rate (C) vs. specific growth rate (*μ*) of the different strains evaluated.** Error bars show the standards deviation between triplicates.

A comparison of the performance of the mutant strains is shown in Figure 2. Since the productivity of a process is given not only by the final concentration obtained, but also by the product yield (Y_p/x_) and specific production rate (*q*_*p*_), such values were compared in relation with the specific growth rate (*μ*) for W3110, VH33 and all mutant strains. It is generally assumed that the specific growth rate of *E. coli* is inversely proportional to Y_p/x_ (for a review on this issue, see [1]). However, such a correlation was not observed by for all the strains studied here. As shown in Figure 2A, strain VH33 grew slower (0.37 ± 0.01 h^-1^) than W3110 (0.61 ± 0.01 h^-1^) and its Y_p/x_ value was 2.4 times higher than that of W3110. Notwithstanding the higher *μ* of W3110, its *q*_*p*_ value was much lower than that of VH33 (Figure 2B). The important difference in specific glucose consumption rate (*q*_*s*_) (Figure 2C) is in agreement with the molecular design of VH33 to reduce overflow metabolism. The deletion of genes related to nucleotide catabolism increased the growth rate of VH33 (up to 32% in the case of VH33 Δ *deoR*), whereas Y_p/x_ remained relatively unchanged (Figure 2A), which in turn resulted in a decrease of *q*_*p*_ (Figure 2B) and was accompanied by a large increase of *q*_*S*_ (Figure 2B). Due to the fact that the biomass yield in glucose (Y_x/s_) did not change for these mutants compared to VH33 (data not shown), it could be hypothesized that glucose was consumed faster in order to synthesize more nucleotides, but it was not reflected in an increase of pDNA production since the regulation of plasmid replication is still present as *recA* gene was not deleted in this strain. As described earlier, the most important changes were seen when *recA* gene was deleted from VH33. The sole deletion of *recA* had a slight effect on growth rate but increased Y_p/x_ in 283%. Consequently, *q*_*p*_ increased considerably, compared to VH33 (from 0.45 ± 0.05 to 1.15 ± 0.09 mg/g h) (Figure 2 A-B). Interestingly, *q*_*s*_ changed slightly as a result of this mutation (Figure 2C), but Y_x/s_ decreased from 0.52 ± 0.01 to 0.43 ± 0.01 g/g, suggesting that more carbon was directed to energy generation necessary for pDNA synthesis.

Although the double mutants VH33 Δ (*recA deoR*) and VH33 Δ (*recA nupG*) did not produce more pDNA than the single mutant VH33 Δ (*recA*), they consumed glucose faster than the other mutants (Figure 2B), probably by the same reason that was proposed above. Slightly higher Y_p/x_ values were observed for the double mutants compared to VH33 Δ (*recA*). However, the Y_x/s_ of the double mutants were also slightly lower (9%) than for VH33 Δ (*recA*) (data not shown), which explains the unchanged final pDNA concentration. Nevertheless, the *q*_*p*_ values of these mutants, particularly VH33 Δ (*recA deoR*) was higher than that of VH33 Δ (*recA*). Finally the triple mutant reached the highest Y_p/x_ (4.12 ± 0.20 mg/g) and *q*_*p*_ (1.46 ± 0.10 mg/g h) values of all the mutant strains. Based on these results, the triple mutant strains VH33 Δ (*recA deoR nupG*) was selected for evaluation in high cell-density cultivations in batch mode.

### Cultivation in small-scale bioreactors

**Figure 3:**
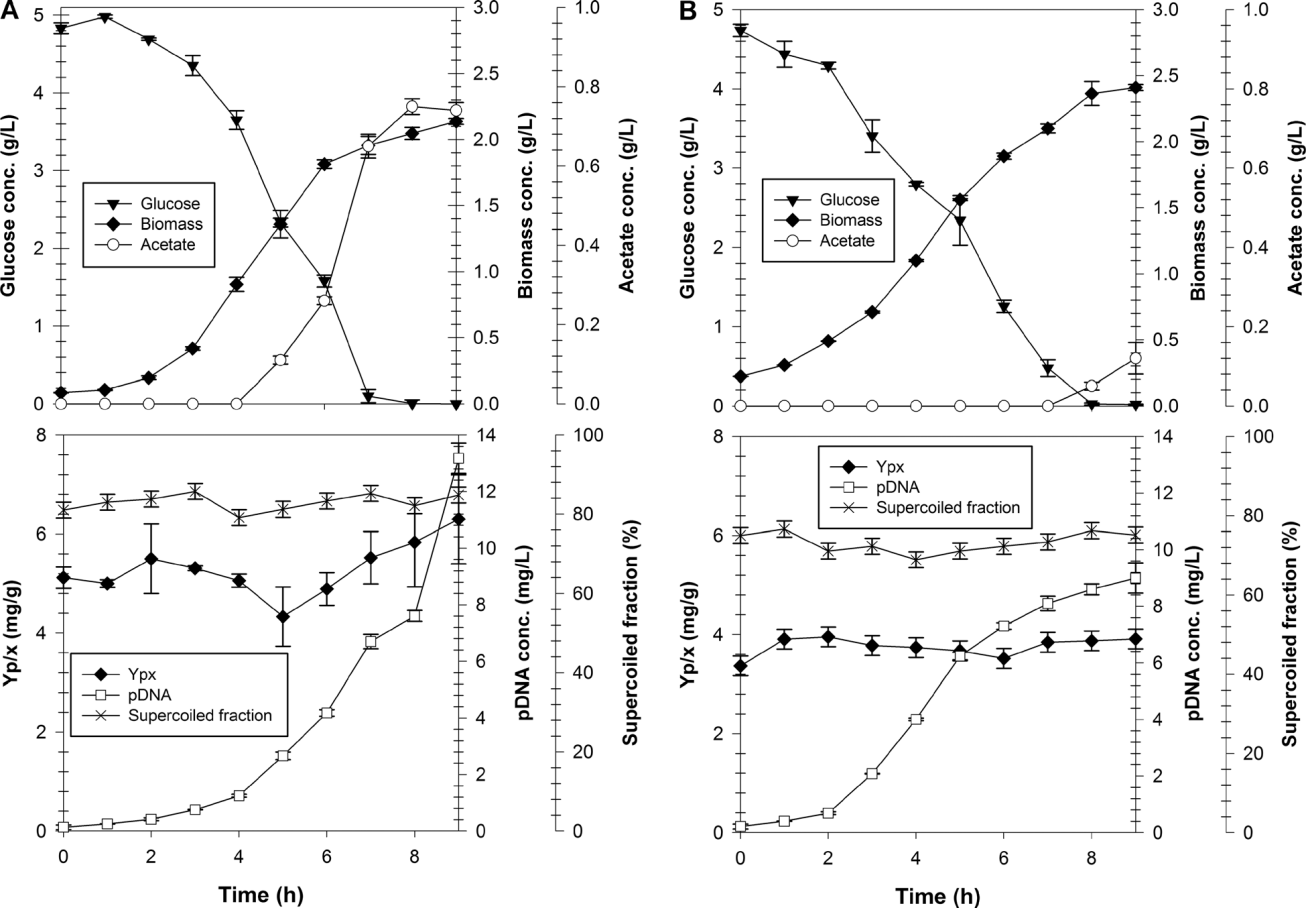
**Growth profile of strains DH5α (A) and VH33 Δ (*recA deoR nupG*) (B) in batch cultivations with an initial glucose concentration of 5 g/L.** Top panels: glucose, biomass and acetate concentrations. Bottom panels: pDNA yield on biomass, pDNA concentration and pDNA supercoiled fraction. Error bars show the standards deviation between duplicates.

The performance of strain VH33 Δ (*recA deoR nupG*) under well defined conditions was evaluated in small-scale bioreactors. Such experiments allowed the attainment of high cell-densities in batch mode, something that cannot be achieved in shake flask due to the lack of pH and dissolved oxygen tension control. Two groups of cultivations were carried out: using low (5 g/L) and high (100 g/L) initial glucose concentrations. For comparison, the commercial strain DH5α was cultivated under the same conditions. Results of cultivations using low initial glucose concentration can be seen in Figure 3. The results of bioreactor cultivation using 5 g/L of initial glucose are similar to those of shake flask: DH5α strain produced 13.09 ± 0.34 mg/L of pDNA and 0.66 ± 0.02 g/L of acetate, which started to accumulate 4 h after inoculation (Figure 3A). The pDNA supercoiled fraction was nearly constant and higher than 80%, and the Y_p/x_ value also remained relatively constant between 5–6 mg/g (Figure 3A). VH33 Δ (*recA deoR nupG*) produced 8.8 ± 0.22 mg/L of pDNA while maintaining a supercoiled fraction close to 80% and a relatively constant Y_p/x_ of around 4 mg/g (Figure 3B). This latter result is interesting since previous results of VH33 showed a decrease of Y_p/x_ throughout the batch cultivation [20]. The plasmid stability in the triple mutant strain is an additional advantage provided by the deletion of *recA*. However, pDNA production was higher in DH5α cultivations under low glucose concentrations.

**Figure 4:**
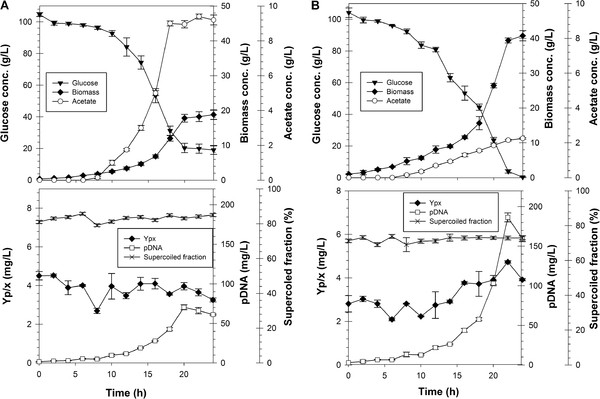
**Growth profile of strains DH5α (A) and VH33 Δ (*recA deoR nupG*) (B) in batch cultivations with an initial glucose concentration of 100 g/**^**L**^**.** Top panels: glucose, biomass and acetate concentrations. Bottom panels: pDNA yield on biomass, pDNA concentration and pDNA supercoiled fraction. Error bars show the standards deviation between duplicates.

The second group of cultivations aimed at attaining high cell-densities in batch mode using an initial glucose concentration of 100 g/L. The growth profile of DH5α at 100 g/L of initial glucose is shown in Figure 4A. As it can be seen, acetate accumulated up to 9.5 ± 0.8 g/L. The growth rate was only 0.17 ± 0.02 h^-1^, which means a decrease of more than 60% compared to conditions of low glucose concentration. Growth ceased at 18 h, when acetate concentration was around 9 g/L and glucose concentration was still above 10 g/L. Growth cessation can be attributed to the elevated acetate concentration, which is known to be toxic for *E. coli* at concentrations of 5 g/L [34]. In consequence, Y_x/s_ was only 0.19 ± 0.03 g/g, which represented a decrease of 50% compared to low glucose concentration cultivations. The pDNA supercoiled fraction was not affected by these conditions (Figure 4A), but the Y_p/x_ value was approximately 30% lower than cultivations with 5 g/L of glucose and decreased throughout the cultivation (Figure 4A). As a result of the low yields and high acetate accumulation, the pDNA produced by DH5α reached only 70 ± 4 mg/L (Figure 4A).

As shown in Figure 4B, strain VH33 Δ (*recA deoR nupG*) produced a relatively low amount of acetate (2.2 ± 0.1 g/L), as expected from its engineered glucose transport system. Noticeably, the specific growth rate decreased to (0.15 ± 0.02 h^-1^), which is a reduction of nearly 50% compared to conditions of low glucose concentration. Such a decrease in growth rate has been observed before [18-20,35] and attributed to the elevated osmolality of the highly concentrated medium. Also, Y_x/s_ decreased to 0.40 ± 0.03 g/g, which represented a reduction of around 17% with respect to low glucose conditions. Yet, high cell-densities were attained, since biomass reached a concentration of 40 g/L. Additionally, the pDNA supercoiled fraction was not significantly affected by these cultivation conditions, since it remained relatively constant at around 75% throughout the cultivation (Figure 4B). In contrast, the Y_p/x_ was close to 3 mg/g during the first 15 h of cultivation, and increased thereafter to around 4 mg/g until the end of the batch, probably due to a decreased osmotic stress due to the lower glucose concentration (60 g/L from 15 h). As a result of the most favorable growth performance, compared to DH5α cultivations (less acetate accumulated), the maximum pDNA concentration reached by VH33 Δ (*recA deoR nupG*) was 186 ± 15 mg/L. Such pDNA concentration is, to the best of our knowledge, the highest ever reported for a batch cultivation of *E. coli*. Some of the highest pDNA concentrations reported for batch mode, are shown in Table 1. As it can be seen, the two highest concentrations attained prior to the present work, employed cultures with very rich media that can either sensibly increase production costs (in the case of amino acids and nucleotide additions) or reduce the reproducibility of the process and generate a considerable amount of foam (in the case of complex media), which can be an important concern in large-scale cultivations. If pDNA yields of VH33 Δ (*recA deoR nupG*) were to be improved, simple additions to the used media, such as glutamate or casaminoacids would be a simple alternative. Additional cell engineering strategies could also be implemented to improve pDNA production.

**Table 1:** Some of the highest pDNA concentrations reached in batch cultivations

**Reference**	**Strain used**	**Cultivation medium**	**Carbon source (concentration)**	**pDNA concentration reached (mg/L)**
[36]	DH5α	Defined, supplemented with glutamate	Glycerol (52 g/L)	45
[37]	JM109	Defined, supplemented with 20 aminoacids and nucleotides	Glucose (5 g/L)	60
[38]	HB101	Complex, supplemented with yeast extract, casaminoacids, torula yeast RNA and RNase A	Glucose (20 g/L)	109
[39]	DH5α	Complex, supplemented with casein peptone and yeast extract	Sucrose (10 g/L) and glycerol (10 g/L)	52
[20]	VH33	Mineral	Glucose (100 g/L)	40
**This work**	VH33 Δ (*recA deoR nupG*)	Mineral	Glucose (100 g/L)	186

## Conclusions

Cultivation of the engineered strain VH33 Δ (*recA deoR nupG*) using high glucose concentrations allowed the attainment of high cell-densities in batch mode and the production of high amounts of pDNA. Further strategies are needed to reduce some undesired effects of high glucose-concentrations, like the reduction in yields and growth rate. Overall, the present study represent a useful option to avoid, through cell engineering strategies, traditional cultivation problems such as overflow metabolism and presence of substrate gradients.

## Materials and methods

### *E. coli* strains and plasmids

*E. coli* W3110 (ATCC 27325), VH33 *(ΔptsH, ΔptsI, ΔlacI, lacZ::loxP)*, DH5α *(endA1, recA*1*, gyrA*96*, thi, hsd*R17*, relA*1*, sup*E44*, ΔlacU*169*,* Φ80*, lacZ*ΔM15) and P1 *vir* phage where laboratory collection material. For assessment of pDNA production, a 6.1 kb plasmid named pHN was used. Plasmid pHN was constructed from the pcDNA3.1(+) plasmid (Invitrogen), which contains the pUC origin of replication and an ampicillin resistance gene. A viral haemagglutinin-neuraminidase gene was cloned under transcriptional control of the cytomegalovirus promoter. pHN plasmid is being evaluated as a DNA vaccine against mumps in humans [40].

### Gene deletions

Gene knock-outs were carried out by recombination using plasmid pKD46 as previously described [41]. Chloramphenicol markers in plasmids were amplified by PCR to knock out *endArecAnupG* and *deoR* genes, respectively, and are reported in Table 2. PCR products carrying antibiotic markers and homologous region (40 bp) were electroporated into *E. coli* W3110 carrying pKD46 where lambda recombinase was fully induced by growth on L-arabinose during cultivation at 30°C. 2 h after electroporation and incubation at 37°C, cells were spread on LB agar plates containing chloramphenicol (30 mg/mL). Among candidate colonies, specific gene disruptions were identified by PCR with primers which can hybridize upstream or downstream of deleted *endArecAnupG* and *deoR* genes, respectively. The primers sequences are depicted in Table 3. The disrupted genes, carrying the drug markers, were transferred to VH33 strain by standard P1 transduction [42]. Gene disruptions in the VH33 strain were reconfirmed by PCR.

**Table 2:** Sequences of the oligonucletides used for chromosomal inactivation

**Gen**	**Oligonucleotide**	**5**^**′**^**-3**^**′**^** Sequence**
***recA***	*recA*1	GTTGCGGCCTAAAGAGACATCTACTCTCGCTTCCGCATCG-ATGGGAATTAGCCATGGTCC
***recA***	*recA*2	CAACAGAACATATTGACTATCCGGTATTACCCGGCATGAC-TGTAGGCTGGAGCTGCTTCG
***endA***	*endA*1	AAGCGCGTTGCACATACGGGTTATGATTGCCCTGCACCTT-CATGGGAATTAGCCATGGTC
***endA***	*endA*2	GGCCCGGCGTTGGCCGAAGGTATCAATAGTTTTTCTCAGG-TGTAGGCTGGAGCTGCTTCG
***nupG***	*nupG1*	ATGTGCTTTTTCAAACACTCATCCGCATCACGATGTGAGG-TGTAGGCTGGAGCTGCTTCG
***nupG***	*nupG2*	TTGAACATCGCCATGAACGCGAAGGCCAGAACCACGGAGT-ATGGGAATTAGCCATGGTCC
***deoR***	*deoR1*	CACGTCGCGAAGAGCGTATCGGGCAGCTGCTGCAAGAATT-TGTAGGCTGGAGCTGCTTCG
***deoR***	*deoR2*	TTTACTGTGGTCGACAACCAGCACATGCTTTTGCGCCATC-ATGGGAATTAGCCATGGTCC

**Table 3:** Sequences of the oligonucleotides used for chromosomal insertions comprobation

**Gen**	**Oligonucleotide**	**5**^**′**^**-3**^**′**^**sequence**
***recA***	*recA*1	ATGGGAATTAGCCATGGTCC
***recA***	*recA*2	TGTAGGCTGGAGCTGCTTCG
***endA***	*endA*1	CGTGGCTGACCAGCTCATCT
***endA***	*endA*2	TGCAGGTCGCTTCACGACTC
***nupG***	*nupG1*	CTTCGCGGATTATCTGCTGA
***nupG***	*nupG2*	GTGGCAGGATTATCCGACAT
***deoR***	*deoR1*	GTCCGGTAATGACGCCTGTA
***deoR***	*deoR2*	CAACGACTTGCCTGTATTGG

### Cultivation media

Cultivation medium had the following composition (in g/L): K_2_HPO_4_, 17; KH_2_PO_4_, 5.3; (NH_4_)_2_SO_4_, 2.5; (NH_4_)Cl, 1; sodium citrate, 1; MgSO_4_·7H_2_O, 1; ampicillin disodium salt, 0.1; thiamine hydrochloride, 0.01 and 2 mL of a stock solution of trace elements [20] per L of medium. The medium was supplemented with 5 or 100 g/L of glucose, which was sterilized separately and added to the cold medium. For shake flasks cultivations, 3-(N-morpholino) propanesulfonic acid (MOPS) was added as a buffer at a final concentration of 20 mM. Ampicillin disodium salt (0.1 g/L) was used as selective pressure in all shake flask and bioreactor cultivations.

### Precultures development

Cryo-preserved *E. coli* cells were cultivated in 250 mL baffled shake flasks containing 50 mL of the described medium, including 0.1 g/L of ampicillin disodium salt and 5 g/L of glucose. Precultures were maintained at 37°C and 200 rpm in an orbital shaker for 18 h. Cells were taken during exponential growth phase, and 100 mL of the preculture were centrifuged at 4000 rpm for 10 min at 4°C. The resulting pellet was resuspended in 10 mL of fresh mineral medium and this concentrated biomass was used to inoculate a 3 L bioreactor. The initial biomass concentrations for low cell-density cultivations (5 g/L of initial glucose) were 0.25 ± 0.1 g/L. In the case of high cell-density cultivations the initial biomass concentration were 2.0 ± 0.2 g/L.

### Shake flask cultivations

Shake flasks cultivations were conducted at 37°C and 200 rpm in an orbital shaker. Samples for glucose and acetate analyses were taken every h. Samples for pDNA analyses were taken at glucose exhaustion. All the cultivations were performed by triplicate. The final pH was above 6.8 for all the cultivations.

### Bioreactor cultivation

*E. coli* strains were cultivated in a BioFlo 110 Modular Fermentor System (New Brunswick Scientific, Edison, NJ) using a set of 3 L bioreactors. A working volume of 1.6 L was used. AFS-Biocommand Bioprocessing Software (New Brunswick Scientific) was used for data logging and ope-rational parameters control. Temperature was set at 37°C and dissolved oxygen tension (DOT) was maintained above 30% with respect to air saturation by increasing stirrer speed (from 200 to 900 rpm) and enriching air with pure oxygen in order to ensure fully aerobic conditions. In addition, gas flow rate was manually varied from 0.75 to 2 vvm when necessary to contend with the high oxygen demand in batch cultures. A 15% NH_4_OH solution was used to control pH to 7.2. Silicone-based antifoaming agent was added on demand. All batch cultures were followed until glucose depletion. All batch cultivations were run by duplicate.

### Analytical methods

Acetate concentration was determined by HPLC as previously described [18]. Glucose concentration was determined off-line with an YSI 2700 biochemical analyzer (Yellow Springs Instruments, Yellow Springs, OH). Dry cell weights were obtained from cells pellet samples dried at 65°C for at least 18 h. pDNA was extracted from 2 mg of wet biomass using the Qiagen Spin Mini Prep kit (Hilden, Germany), following the instructions of the manufacturer and eluting the pDNA in 70 μL of TE buffer at 70°C. DNA concentration was measured spectrophotometrically at 260 nm using a Nanodrop UV spectrophotometer ND-1000 (NanoDrop, Willmington, DE). The pDNA supercoiled fraction was analyzed by image analyses of agarose gels electrophoresis as described earlier [20].

## Competing interests

The authors declare that they have no competing interests.

## Authors’ contributions

GMB and EMM carried out the mutations. GMB also carried out the cultivation experiments. BB, GG and OTR contributed to the design of experiments and interpretation of data. ARL conceived the study, participated in its design and coordination and in the drafting of the manuscript. All the authors read and approved the final manuscript.
